# NAIRDB: a database of Fourier transform infrared (FTIR) data for nucleic acids

**DOI:** 10.1093/nar/gkae885

**Published:** 2024-10-16

**Authors:** Elsa Balduzzi, Frédéric Geinguenaud, Dominik Sordyl, Satyabrata Maiti, Masoud Amiri Farsani, Grigory Nikolaev, Véronique Arluison, Janusz M Bujnicki

**Affiliations:** Laboratoire Léon Brillouin, UMR 12 CEA/CNRS, Bâtiment 563, Site de Saclay, 91191 Gif-sur-Yvette, France; Université Sorbonne Paris Nord, Université Paris Cité, Laboratory for Vascular Translational Science, LVTS, INSERM, UMR 1148, 74 rue Marcel Cachin, F-93017 Bobigny, France; Laboratory of Bioinformatics and Protein Engineering, International Institute of Molecular and Cell Biology in Warsaw, ul. Ks. Trojdena 4, PL-02-109 Warsaw, Poland; Laboratory of Bioinformatics and Protein Engineering, International Institute of Molecular and Cell Biology in Warsaw, ul. Ks. Trojdena 4, PL-02-109 Warsaw, Poland; Laboratory of Bioinformatics and Protein Engineering, International Institute of Molecular and Cell Biology in Warsaw, ul. Ks. Trojdena 4, PL-02-109 Warsaw, Poland; Laboratory of Bioinformatics and Protein Engineering, International Institute of Molecular and Cell Biology in Warsaw, ul. Ks. Trojdena 4, PL-02-109 Warsaw, Poland; Laboratoire Léon Brillouin, UMR 12 CEA/CNRS, Bâtiment 563, Site de Saclay, 91191 Gif-sur-Yvette, France; Université Paris Cité, UFR SDV, 35 Rue Hélène Brion, 75013 Paris, France; Laboratory of Bioinformatics and Protein Engineering, International Institute of Molecular and Cell Biology in Warsaw, ul. Ks. Trojdena 4, PL-02-109 Warsaw, Poland

## Abstract

The Nucleic Acid InfraRed Data Bank (NAIRDB) serves as a comprehensive public repository dedicated to the archival and free distribution of Fourier transform infrared (FTIR) spectral data specific to nucleic acids. This database encompasses a collection of FTIR spectra covering diverse nucleic acid molecules, including DNA, RNA, DNA/RNA hybrids and their various derivatives. NAIRDB covers details of the experimental conditions for FTIR measurements, literature links, primary sequence data, information about experimentally determined structures for related nucleic acid molecules and/or computationally modeled 3D structures. All entries undergo expert validation and curation to maintain the completeness, consistency and quality of the data. NAIRDB can be searched by similarity of nucleic acid sequences or by direct comparison of spectra. The database is open for the submission of the FTIR data for nucleic acids. NAIRDB is available at https://nairdb.genesilico.pl.

## Graphical Abstract

**Graphical Abstract:**
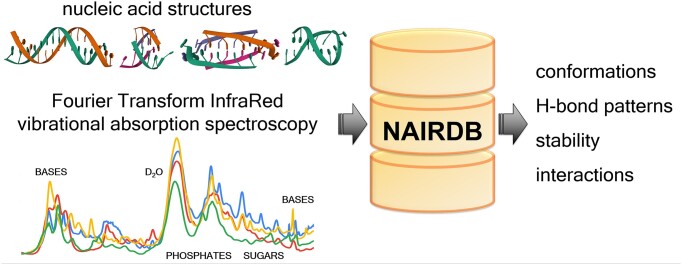

## Introduction

Fourier transform infrared (FTIR) spectroscopy is a powerful analytical technique used to study the vibrational characteristics of molecules. It operates based on the interaction between a sample and infrared (IR) radiation that, within few exceptions, is not energetic enough to allow an electronic transition as in ultraviolet (UV) absorption spectroscopy [including circular dichroism (CD)] but induces vibrational transitions. The vibrational energy absorbed is related to the force constant (*k*) of the bond and the reduced mass of the atoms involved, according to Hooke’s law. The position and intensity of the band are strongly influenced by the force constant of the covalent bond and variations in the electric dipole moment during vibration. For example, highly polarized C=O, P=O and C–O–C bonds exhibit strong absorption bands. Compared with UV spectra, nucleic acids (NAs) have numerous IR absorption bands, most of which cannot be attributed to a single bond due to vibrational coupling. In particular, the C_2_=O_2_, C_4_=O_4_ and ring C_5_=C_6_ vibrations are highly coupled in thymine and uracil, and several other ring, carbonyl and NH bending vibrations are coupled in each of the nucleobases (1,2). Further, the origins of spectral changes upon base pairing involve vibrational coupling within the base pair and are still difficult to model (3–5). Although vibrational coupling within NAs presents challenges, FTIR spectroscopy remains a valuable tool for identifying functional groups and observing changes in the NA structure, including those caused by hydrogen bonding and other interactions where dipole moment changes during vibration, which significantly influence the covalent bond vibration.

While most spectrometers use dispersive elements such as gratings or prisms to isolate wavelengths, the cornerstone of FTIR spectrometer is the Michelson interferometer. Based on a specific configuration of mirrors and on wave interference, the interferometer allows the alteration of light frequencies and the simultaneous measurement of a wide range of wavelengths. Nevertheless, in this setup, the raw data (called the ‘interferogram’, i.e. light intensity as a function of the moving mirror position) must be converted into a spectrum (light absorption as a function of frequency). This is where the Fourier transform converts the displacement of the mirror (in cm) into its inverse domain, the wavenumbers (in cm^−1^). In IR spectra (in general vibrational spectroscopy), the wavenumber expressed in cm^−1^ is commonly used rather than the wavelength in nm.

The FTIR spectrum provides a unique fingerprint of a sample, enabling the identification and characterization of the molecular structure and functional moieties of the analyzed molecular system, based on its vibrational transitions. FTIR spectroscopy is widely used across various scientific fields, including chemistry, materials science, forensic or medicine. In biology, it is particularly valuable for studying the structural characteristics of biological macromolecules. Although FTIR spectroscopy has been used for years to analyze protein conformation (6), its application to NAs is less frequent but particularly instructive. The FTIR spectra allow the acquisition of rich information about NA structure, including base pairing, sugar conformation or glycosidic torsional angle (7). While FTIR spectroscopy offers the advantage of being label-free, it has a potential for incorporating nonperturbative, site-specific labels using isotope editing. Isotope labeling, such as ^13^C and ^15^N, has been used to study the labeled guanine residue at the central position [d(T-GG-G*-G-G-T)] to study the structure of G-quadruplexes (8). Furthermore, FTIR requires relatively small amounts of sample, and allows for easy changes in experimental conditions such as pH, solvent or temperature.

FTIR analyses are often combined with other IR spectroscopic techniques, such as temperature jump, which focuses on the dynamic response to rapid heating at specific wavelengths, and 2D IR, which uses multiple IR pulses to explore interactions between NA vibrational modes and their dynamics. FTIR has also been combined with other experimental techniques such as UV optical melting experiments, Raman spectroscopy, nuclear magnetic resonance spectroscopy, CD spectrometry and isothermal titration calorimetry, and with computational modeling of molecular structures, e.g. molecular dynamics. Recently, the use of IR spectroscopy has been extended to single molecule detection, with IR coupled to atomic force microscopy (AFM-IR or nanoIR) emerging as a tool for the investigation of single NA or nucleoprotein complex at the nanoscale (9).

FTIR was used to monitor the reversible B- to A-form conformational transitions of DNA, in response to dehydration and rehydration, both *in vitro* (10) and in eukaryotic cells (11) [reviewed in (12)]. It was also used to provide insights into the interactions of NAs with metal ions (13,14). FTIR combined with time-resolved IR spectroscopy was used to examine transfer RNA folding dynamics, highlighting how rapid thermal changes influence RNA structures (15). Recently, a combination of different techniques, including FTIR, was used to study the effects of abasic sites on the thermodynamic stability, kinetics, base-pair dynamics and cooperativity of helix formation for DNA duplexes (16) and to analyze non-nearest-neighbor effects arising from the positional dependence of incorporating abasic sites in DNA (17). It was also used to measure the thermodynamics and kinetics of DNA and RNA dinucleotide hybridization to gaps in DNA and RNA helices (18). FTIR spectroscopy has been used to analyze the effects of chemical modifications (19) and sequence variation (20,21) on NA structures. FTIR has been beneficial in studying the structural effects of NA interactions with proteins (22). FTIR was also used to study the cellular content of DNA and RNA, particularly the DNA content and ploidy (23) and to detect variations in cellular RNA levels that correlate with cellular metabolic activities (24,25).

**Figure 1.**
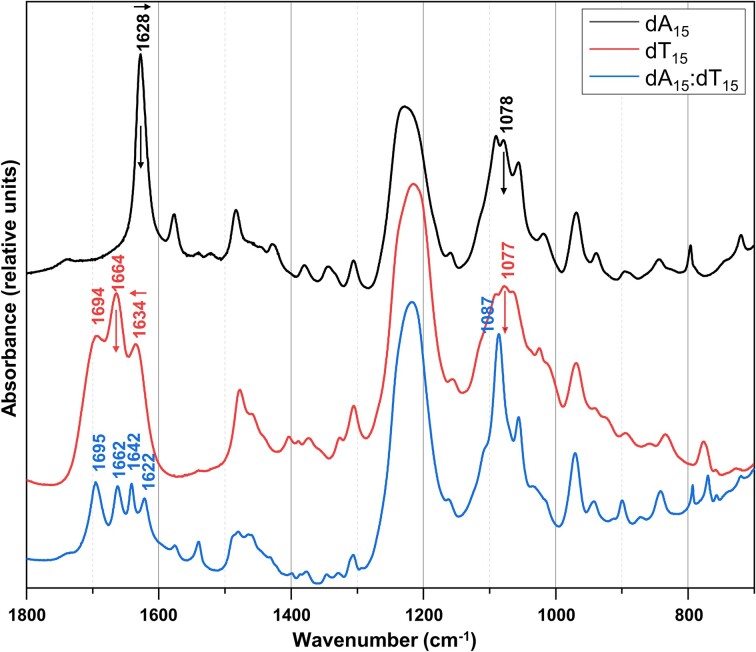
Typical FTIR NA spectra in D_2_O. Five main regions can be used to analyze the NA sugar moiety: out-of-plane base vibrations spectral domain (∼800–760 cm^−1^ range); sugar conformation spectral domain (∼900–800 cm^−1^ range); antisymmetric phosphate spectral domain (∼1250–1150 cm^−1^ range); symmetric phosphate spectral domain (∼ 1080–1090 cm^−1^ range); and in-plane base vibrations spectral domain (∼1750–1450 cm^−1^ range). For instance, the band at 1628 cm^−1^ (upper spectrum) corresponds to the vibration of ND_2_ coupled to ring of A (in deuterated buffer). The bands at 1694, 1664 and 1634 cm^−1^ (middle spectrum) imply C_2_=O_2_, C_4_=O_4_ and ring of T in single strand dT_15_, even if IR absorption bands often cannot be attributed to a single bond due to vibrational coupling. The band at 1642 cm^−1^ (lower spectrum) corresponds to T-ring vibration in double-stranded dA_15_-dT_15_. FTIR can also probe contacts on base and H-bond formation, which may induce either a shift in band position usually to a lower wavenumber (compare upper and lower spectra at 1628 cm^−1^ versus 1622 cm^−1^) or a decrease in band intensity (compare middle and lower spectra at 1664 cm^−1^).

Despite the obvious utility of FTIR data and numerous FTIR studies on NAs reported in the literature, no specific data resource has been made available to the scientific community to present FTIR spectra and associated metadata. Thus far, few analyses have been made aiming to synthesize the data available in the literature. One notable effort in this direction was the development of a library of IR bands of NAs in solution (26,27), which presented a comprehensive compilation of IR bands characteristic of NAs in various conformations. The bands cover the mid-IR spectra ranging from 1800 to 800 cm^−1^, and are detailed in five main spectral regions (Figure 1), each highlighting specific structural features such as base pairing and stacking, sugar pucker and backbone conformations. They are helpful for identifying specific NA interactions and conformations, offering insights into changes in structure induced by environmental factors or binding interactions.

Despite the accumulation of FTIR analyses of NAs, a comprehensive database of FTIR spectra has not been compiled until now. Such a resource would benefit the scientific community by facilitating easier access to and interpretation of FTIR data in NA research. The Nucleic Acid Infrared Database (NAIRDB) aims to fill the gap between the need for systematic information on the FTIR of NAs and the dispersed information on that topic. NAIRDB contains an extensive collection of FTIR spectra for a wide range of NA molecules, including DNA, RNA and their derivatives. The NAIRDB database provides a systematic collection of FTIR datasets, with detailed descriptions of experimental conditions, including sample preparation, data acquisition parameters and data processing methods. The database also includes predicted secondary and tertiary structures of the NAs, wherever such predictions can be reliably generated, and literature references for each dataset.

The NAIRDB database provides a valuable resource for researchers interested in the structure and function of NAs and can be used for comparative analyses of NA structures across a wide range of experimental conditions. It can be used in conjunction with CD analysis (28), using NACDDB, the Nucleic Acid Circular Dichroism Database (29). While UV absorbance and CD are sensitive to electronic transitions and chiral structures, FTIR spectroscopy provides unique sensitivity to molecular vibrations, which directly reflect local interactions such as hydrogen bonding and changes in the electrostatic environment arising from functional groups. Vibrational energy depends on the force constant, so alterations in hydrogen bonding within base pairs or shifts in electrostatic conditions affect vibrational frequencies, offering detailed insights into NA conformation. Unlike UV and CD, FTIR can detect these subtle changes, complementing CD’s use in studying RNA thermal stability.

## Database content

The current version of NAIRDB (as of 13 September 2024) contains 202 entries, and this dataset is expected to grow with the planned updates. NAIRDB presents spectra recorded in either water (H_2_O) or heavy water (D_2_O). It should be emphasized that water has a very active vibration in the base region around 1645 cm^−1^ (also corresponding to the protein amide I region), which can mask the signal of the macromolecule. Deuterated buffers are thus currently used to analyze base vibration. Nevertheless, D_2_O has also a vibration at 1210 cm^−1^, the region of phosphate vibration. The use of hydrogenated and deuterated solvents is thus complementary, and both are presented in NAIRDB.

Two main types of vibrational modes are observed in IR spectroscopy: (i) stretching, which involves the elongation of the bond analyzed (stretching can be symmetrical or asymmetrical); and (ii) bending and in general other deformation vibrations, which correspond to changes in the connection angles (shearing, tilting, out-of-plane agitation and twisting). Thus, some bands are characteristic of the conformation of the NA backbone, while others are characteristic of the nucleotide or base pair. Both are detailed in NAIRDB. As examples of conformational bands, a B-form helix shows a band corresponding to asymmetric vibration of phosphate around 1225 cm^−1^, while for A-helix this band appears around 1240 cm^−1^ and for Z-helix around 1215 cm^−1^.

For specific parts of nucleotide sugars, bands between 842 and 820 cm^−1^ are characteristic of the S-type (C2′ endo), while bands around 880, 860 and 815 cm^−1^ correspond to N-type (C3′ endo) sugar. Each type of base and consequently base pair also has characteristic bands. For example, when guanine is engaged in an H-bond, a band around 1689–1678 cm^−1^ is observed, while for free guanine, bands observed are rather between 1673 and 1660 cm^−1^. Such a vibration is particularly useful to detect the formation of quadruplexes and whether the G-quartet formed is parallel (with a band around 1693 cm^−1^) or antiparallel (the band is then around 1682 cm^−1^).

### Acquisition and treatment of FTIR spectra

FTIR can be performed in two different operational modes. In the transmission mode, the sample consists of a thin film with a thickness of around 50 μm. However, since water shows strong IR absorbance, it can easily saturate the detector. To avoid this problem, D_2_O is generally used in transmission mode. In the reflection mode, an attenuated total reflectance (ATR) setup with a crystal is used to generate an evanescent wave that penetrates the sample to a depth of a few micrometers. However, because the evanescent wave’s penetration depth is wavelength-dependent, some corrections must be applied to ATR-FTIR spectra. Additionally, since ATR is a surface technique, it might lead to various artifacts, and hence, we decided not to include any ATR spectra in the current version of the database; however, we are open to include such spectra in the future.

There are two types of spectra in the current version of the database: (i) Samples in solution (e.g. NAIRDB entry ID 88, polydA-polydT_D2O) that were prepared to have the absorbance of ∼3.5 optical density (OD). They were deposited on a ZnSe window and dried, followed by the deposition of 1 μl of solvent. (ii) Samples that were dried and then placed in a container, where the relative humidity of the atmosphere was controlled to obtain the desired hydration level (e.g. NAIRDB entry ID 94, polydA-polydT_H2O_RH100). The detailed protocol is described in (10).

Spectra were recorded for wavelengths ranging from 4000 to 400 cm^−1^; however, only the wavelength ranging from 1800 to 700 cm^−1^ is presented in NAIRDB, as this is the region where the information for NAs is found. The resolution was 1 cm^−1^ for all the spectra. No solvent subtraction was performed. Raw spectra were converted to absorbance and interpolated between 1800 and 700 cm^−1^ with an interval of 0.2 cm^−1^. Spectra were then normalized by dividing the spectrum by the area under the curve of the phosphate band (symmetric stretching vibration around 1090 cm^−1^). Automatic zeroing was then applied using the lowest absorbance value. This process was applied so that spectra can be compared with each other. Detailed protocols for recording NA FTIR spectra can be found in (7).

## Implementation

The database was developed with the Django web framework (version 4.2.13) and is hosted on an HTTP server powered by Nginx (https://www.nginx.com/). To ensure the secure transmission of sensitive information, NAIRDB employs the HTTPS protocol, encrypting all data exchanged between the server and the client. The database relies on the SQLite relational database to store and query data. For displaying the database content, open-source JavaScript libraries were implemented. The DataTables library (http://datatables.net) provides tables that support data sorting and searching.

FTIR spectra are visualized using the JavaScript-based Plotly library (https://plotly.com/javascript), which allows users to zoom in on specific parts of the spectra and download plots as ‘.png’ files. Spectra data can also be downloaded in .xlsx format, enabled by the implementation of SweetAlert2 (https://sweetalert2.github.io/). Additionally, the database offers the tertiary structure of the molecules in downloadable pdb-formatted files.

**Figure 2.**
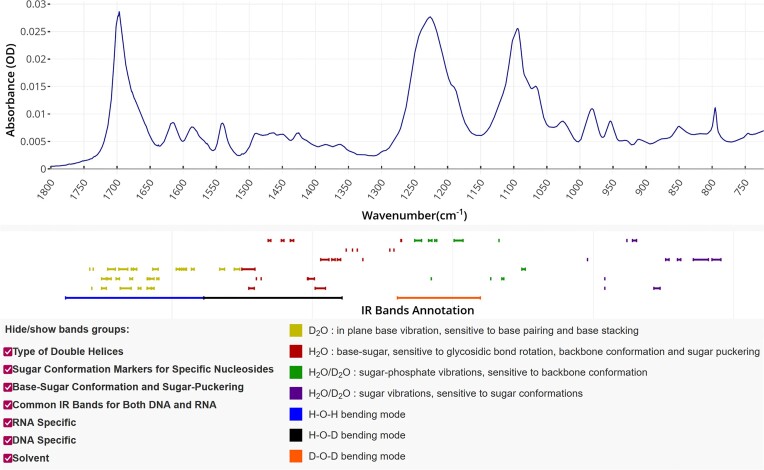
Example entry in NAIRDB: a view on the top panel for experiment ID dG12-18_D2O, FTIR spectrum for a parallel G-quadruplex in D_2_O. IR band annotation lines indicate (from top to bottom) (i) type of double helices, (ii) sugar conformation markers for specific nucleosides, (iii) base–sugar conformation and sugar puckering, (iv) bands common to DNA and RNA, (v) RNA-specific bands and (vi) DNA-specific bands. When overlapping peaks are observed, they can be attributed by considering the sequence of the NA, its propensity to form a specific structure or other techniques allowing the identification of the structure.

Spectra data can be downloaded in .xlsx format, as generated by SweetAlert2 (https:/sweetalert2.github.io/). FTIR spectra are visualized using JavaScript-based Plotly library (https://plotly.com/javascript). It allows users to download the plots as the ‘.png’ file and to zoom in on the interesting parts of the spectra. The annotation of FTIR spectra is displayed below each spectrum, with bands categorized into six groups. These groups can be shown independently for easier identification. Figure 2 shows an example entry in NAIRDB.

NAIRDB provides the predicted tertiary structure of the NA molecules in the form of downloadable pdb- and mmcif-formatted files. At the time of the publication of the database, 50 entries had an experimentally determined structure available for a similar molecule in the RCSB PDB database (30), and for 140 entries, 3D models have been generated by predictive methods, including AlphaFold3 (31) and SimRNA (32), and their local geometry was optimized by QRNAS (33). Visualization of the 3D structures of the analyzed molecules is provided by Mol* Viewer (https://molstar.org/).

The database supports different query approaches. Keyword search enables users to search for phrases and their contextual matches within the database. BLAST+ 2.12.0 is used for sequence similarity comparisons with the BLASTN tool (34,35). Users can refine their search results by adjusting the e-value threshold (default is 1e−02). The matches are displayed in a table format, showing NAIRDB IDs, degrees of similarity and alignment e-values. Results can be downloaded in two formats: tabular or alignment .afa format. Spectra present in the database can be compared with each other, as well as with user-specified data, through the calculation of root mean square deviation between pairs of spectra.

## Discussion

The NA structural biology community needs a comprehensive database that archives and organizes available FTIR data while accepting public deposition and distribution of spectral references, including metadata. NAIRDB currently provides 202 entries, encompassing DNA, RNA and their hybrids. It offers a single entry point for data that, until now, could only be obtained by meticulously analyzing many disparate sources—often difficult to access, such as supplementary materials of published papers or chapters in old books available only in libraries and not online. The database offers users an easy-to-use web interface with the flexibility to browse lists of FTIR experiments and publications, filter results according to user-defined criteria and select the order in which results are displayed. The database can be queried by keywords, and entries can be searched based on NA sequence similarities or FTIR spectra obtained.

We encourage users to contribute to the database by submitting their FTIR data, following standardization and normalization to ensure consistency with the currently deposited spectra. We are open to including data on novel types of molecules, such as locked nucleic acids (36). Future updates of the NAIRDB database may include nanoIR measurements, which have recently emerged for the FTIR investigation of NAs and nucleoprotein complexes at the nanoscale (9) and other types of vibrational spectra, such as Raman and 2D IR spectra. Users are encouraged to use the ‘Contact Us’ tab to communicate new IR spectra for submission. NAIRDB is also open to user feedback, and we particularly encourage users to report any errors or omissions.

The new database empowers researchers in molecular biology, biophysics and bioinformatics to systematically study FTIR spectra that provide important information about NA structures and protein–NA interactions, integrating these data with other complementary datasets. NAIRDB facilitates comparative analyses of the spectra and the development of new predictive tools, such as those for identifying specific NA structural features from newly acquired spectra. Envisaged areas of future application for NAIRDB include identifying general patterns that emerge from the data, such as major bands that distinguish RNA, DNA and various naturally or synthetically modified NAs, differences between single- and double-stranded DNAs, characterizing the level of structural order and disorder in RNA molecules and differentiating NA conformations under varying conditions, such as hydration and salinity levels. The database has the potential to significantly impact the field, as the development of spectroscopic maps for nucleotide vibrations will require access to numerous experimental spectra for benchmarking. In the future, NAIRDB may also contribute to the development of new methods to predict NA structure using experimental data.

## Data Availability

The web interface to the database is available at https://nairdb.genesilico.pl. This website is free for academic (noncommercial) applications, open to all users and no login or password is required.
